# Study on the Crystallization Behavior of Neodymium Rare-Earth Butadiene Rubber Blends and Its Effect on Dynamic Mechanical Properties

**DOI:** 10.3390/ma17010256

**Published:** 2024-01-03

**Authors:** Xiaohu Zhang, Wenbin Zhu, Xiaofan Li, Xinzheng Xie, Huan Ji, Yanxing Wei, Jifu Bi

**Affiliations:** 1 Centers for Aircraft Science, Huangpu Institute of Materials, Guangzhou 510700, China; zhuwenbin@ciac.ac.cn (W.Z.); lixiaofan@ciac.ac.cn (X.L.); xiexinzheng@ciac.ac.cn (X.X.); jihuan@ciac.ac.cn (H.J.); weiyanxing@ciac.ac.cn (Y.W.); 2 Changchun Institute of Applied Chemistry, Chinese Academy of Sciences, Changchun 130102, China

**Keywords:** neodymium butadiene rubber, blended rubber, crystallization behavior, dynamic mechanical properties

## Abstract

Utilizing neodymium-based butadiene rubber as a baseline, this study examines the effect of eco-friendly aromatic TDAE oil, fillers, and crosslinking reactions on neodymium-based rare-earth butadiene rubber (Nd-BR) crystallization behavior. The findings suggest that TDAE oil hinders crystallization, resulting in decreased crystallization temperatures and heightened activation energies (*E*_a_). The crystallization activation energies for 20 parts per hundreds of rubber (PHR) and 37.5 PHR oil stand at −116.8 kJ/mol and −48.1 kJ/mol, respectively, surpassing the −264.3 kJ/mol of the unadulterated rubber. Fillers act as nucleating agents, hastening crystallization, which in turn elevates crystallization temperatures and diminishes *E*_a_. In samples containing 20 PHR and 37.5 PHR oil, the incorporation of carbon black and silica brought the *E*_a_ down to −224.9 kJ/mol and −239.1 kJ/mol, respectively. Crosslinking considerably restricts molecular motion and crystallization potential. In the examined conditions, butadiene rubber containing 37.5 PHR oil displayed no crystallization following crosslinking, albeit crystallization was discernible with filler inclusion. Simultaneously, the crystallinity level sharply declined, manifesting cold crystallization behavior. The crosslinking process elevates *E*_a_, while the equilibrium melting point ($T_{m}^{0}$) noticeably diminishes. For instance, the $T_{m}^{0}$ of pure Nd-BR is approximately −0.135 °C. When blended with carbon black and silica, the $T_{m}^{0}$ values are −3.13 °C and −5.23 °C, respectively. After vulcanization, these values decrease to −21.6 °C and −10.16 °C. Evaluating the isothermal crystallization kinetics of diverse materials via the Avrami equation revealed that both the oil and crosslinking process can bring about a decrease in *n* values, with the Avrami index *n* for various samples oscillating between 1.5 and 2.5. Assessing the dynamic mechanical attributes of different specimens reveals that Nd-BR crystallization notably curtails its glass transition, marked by a modulus shift in the transition domain and a decrement in loss factor. The modulus in the rubbery state also witnesses a substantial augmentation.

## 1. Introduction

Neodymium-based rare-earth butadiene rubber (Nd-BR) exhibits distinct characteristics, including a high cis content, a well-defined chain structure, and a narrow molecular weight distribution. During the stretching process, it readily undergoes strain-induced crystallization, leading to remarkable wear resistance, resilience, and mechanical properties. These attributes render it well suited for the production of high-performance tires with low rolling resistance [1,2,3].

However, the regular molecular weight structure, while contributing to its exceptional properties, also imparts enhanced crystallization capabilities at lower temperatures, resulting in suboptimal performance in cold conditions. For example, Nd-BR can crystallize within the temperature range of −18 to −30 °C, leading to increased permanent deformation under low-temperature compression, reduced elasticity, and, consequently, impacting its effectiveness in low-temperature applications. The study of butadiene rubber’s crystallization properties holds practical significance.

Research conducted by Wu Yixian et al. [4] focused on investigating the isothermal crystallization kinetics and morphology of Nd-BR. Their findings suggest that Nd-BR crystallizes by forming three-dimensional spherulites, with the size of these spherulites increasing in proportion to the cis content. The Avrami index *n* value, during the isothermal crystallization process, falls within the range of 2 to 3. Vu Anh Doan et al. [5] examined the influence of silica on BR crystallization and found that silica accelerates BR crystallization, increasing the degree of crystallinity even after the crosslinking of BR. However, silica has minimal impact on the Avrami index *n* value. Maria et al. [6] proposed that the primary factor determining the crystallization rate is the appearance of non-uniform structures responsible for nucleation, with their activity contingent on their structure and quantity. Further studies, such as the investigation by Wrana C et al. [7], explored the relationship between BR crystallization rate and temperature. It was observed that below −50 °C, the crystallization rate accelerates with rising temperature, while above −50 °C, it decreases with increasing temperature. Li Shengtian et al. [8] reported on the influence of oil content on the processing and crystallization properties of rare-earth butadiene rubber. They noted that an increase in paraffinic oil content results in an extended semi-crystalline time, but its effect on the Avrami index *n* value is minimal.

Kenji Saijo et al. [9] delved into the crystallization behavior of crosslinked polybutadiene, investigating the impact of temperature and orientation on crystallization. Their work revealed a crystallization phenomenon akin to spinodal in oriented crosslinked polymers. Cai Jiali et al. [10] examined the non-isothermal crystallization kinetics of trans 1,4-polybutadiene, determining the average crystallization activation energies for the hexagonal phase and the monoclinic phase of TPBD to be −165.8 kJ/mol and −220.5 kJ/mol, respectively. Additionally, various crystalline structures may form during BR crystallization. Maria Laura Di Lorenzo highlighted that after butadiene rubber crystallization, a three-phase structure exists when cooled to the glass transition temperature, leading to distinct melting behaviors [11]. Xu Yang et al. [12] conducted a systematic investigation into the phase structure of butadiene rubber during low-temperature crystallization, providing insights into nucleation mechanisms and morphology at different temperatures. Wunde [13] studied the effects of filler and other rubbers on the thermally induced crystallization of high-cis BR by dynamic mechanical analysis (DMA) and DSC and found that carbon black (CB) supports the nucleation and growth of BR crystals while styrene-butadiene rubber (SBR) reduces the crystallization of BR in a BR/SBR blend. Sun and Zhang [14] studied the effect of styrene–butadiene–styrene triblock copolymer (SBS) on non-isothermal crystallization kinetics and melting behavior of syndiotactic 1,2-polybutadiene and found that the SBS suppressed the crystallization behavior of s-PB with decreased crystallization rate. Yao [15] studied the phase structure and crystallization behavior of polyethylene in its blends with cis-1,4-butadiene rubber by DSC, scanning electron microscopy (SEM), and optical microscopy, and found that the PE is finely dispersed in the BR matrix, resulting in confined crystallization of PE at relatively low temperature. The crystallization ability of PE increases with domain size, while the confined crystallization decreases.

In light of the comprehensive attention garnered by the crystallization and application of Nd-BR, our prior research delved into the effects of diluent materials on the crystallization kinetics and thermal behavior of butadiene rubber [16]. We also explored the relationship between crystalline melting behavior and crystal structure. In this current study, our focus centers on examining the impact of oil, fillers, and crosslinking networks in blended rubber on crystallization behavior. This investigation holds significant relevance for butadiene rubber product development.

## 2. Materials and Methods

### 2.1. Raw Materials

Neodymium-based butadiene rubber, specifically grade BR 9101N (density: 0.92 g/cm^3^; *M*_n_: 93,380 g/mol, molecular weight distribution (MWD): 3.719), was procured from Dushanzi Petrochemical Co., Ltd. (Karamay, China). In addition, an environmentally friendly aromatic TDAE oil (density: 0.953 g/cm^3^; carbon type distribution for aromatic/naphthenic/paraffinic is 23%/39%/38%) was sourced from Hansheng Chemical Co., Ltd. (Ningbo, China), while silica of grade 7000 GR was obtained from Evonik Degussa (Essen, Germany). Carbon black (CB), available in N330 and N234 grades, was supplied by Cabot (Tianjin, China). The coupling agent bis-[triethoxysilylpropyl] tetrasulfide (TESPT) was procured from Nanjing Rongan Chemical Technology Co., Ltd. (Nanjing, China). Sulfur: HANSUN HXC-2 was procured from Guangdong Hanxinwei New Material Co., Ltd. (Zhongshan, China). Zinc oxide (ZnO) was procured from Weifang Orlon Zinc Industry Co., Ltd. (Weifang, China). 2-benzothiazolesulfenamide, n-cyclohexyl (CBS) was procured from Kemai Chemical Co., Ltd. (Tianjin, China). 1,3-Diphenylguanidine (DPG) was procured from Kemai Chemical Co., Ltd. (Tianjin, China). Stearic acid was procured from Nantong Cata Chemical Technology Co., Ltd. (Nantong, China).

### 2.2. Sample Preparation

Based on tire formulations, two compounds were prepared with the following composition, as shown in Table 1.

Method for preparing TDAE-filled BR rubber: A total of 200 g of rubber was plasticized on a mill at a temperature of 50 °C. Once the rubber was uniformly sheeted out between the rolls, TDAE oil was incrementally added at proportions of 20 parts per hundreds of rubber (PHR) and 37.5 PHR. After each addition, thorough blending was carried out to ensure uniformity, and samples were collected for retention testing. The resulting rubbers were labelled as BR100 (pure Nd-BR), BR-20T (Nd-BR with 20 PHR TDAE oil), and BR-37.5T (Nd-BR with 37.5 PHR TDAE oil).

Method for preparing Nd-BR/CB rubber: Nd-BR, CB N330, zinc oxide, and stearic acid were added to a micro internal mixer (model: XSS-200, Shanghai KeChuang Rubber & Plastic Machinery Co., Ltd., Shanghai, China) at 50 °C and 60 RPM for 2 min. Subsequently, TDAE oil and other additives were added, and the mixing was extended for an additional 3 min before sheeting out the blend. The well-mixed rubber was then uniformly combined with the vulcanizing agent (sulfur and CBS) using a mill. Vulcanized samples were prepared using a flat vulcanizing machine under a pressure of 15 MPa at 160 °C for a duration of 20 min.

Method for preparing Nd-BR/silica rubber: Nd-BR, CB N234, silica 7000 GR, and silane coupling agent TESPT were added to a micro internal mixer (model: XSS-200, Shanghai KeChuang Rubber & Plastic Machinery Co., Ltd., Shanghai, China) at 50 °C and 60 revolutions per minute (RPM) for 2 min. Subsequently, TDAE oil, SA, and ZnO were introduced, and further mixing was carried out. Afterward, the rubber blend was cooled from the mill (model: LRMR-S-150, Shenyang Fairman Detection Instrument Co., Ltd., Shenyang, China) and placed in storage for at least 4 h. Then, it was mixed for an additional 3 min in the internal mixer at 150 °C and 40–60 RPM, in order to finish the salinization reaction. Care was taken to maintain the material temperature below 155 °C during this process. The resulting blend was then cooled and sheeted out, followed by uniform mixing with sulfur, CBS, and DPG using a mill. Vulcanized samples were prepared using a flat vulcanizing machine (model: GT-7014-H50, Gotech Detection Instrument Co., Ltd. (Dongguan, China)) under a pressure of 15 MPa at 160 °C for a duration of 20 min.

### 2.3. Testing and Characterization

Crystallization Behavior Test:

Protocol 1: A differential scanning calorimeter (DSC, Q25, TA Instruments, New Castle, DE, USA) was employed for this test. The samples underwent cooling from 30 °C to −80 °C at a controlled rate of 5 °C/min, followed by an isothermal hold at −80 °C for 1 min. Subsequently, the samples were heated back to 30 °C, with a heating rate of 10 °C/min.

Protocol 2: In this protocol, the samples were subjected to a cooling process from 30 °C to −40 °C at a rate of 10 °C/min. After reaching −40 °C, an isothermal hold of 10 min was maintained, and then the temperature was raised to a designated point, *T*_0_ = −50 °C, at a rate of 10 °C/min. A 1 min isothermal hold was introduced to melt the crystals. This procedure was iteratively conducted with subsequent heating to (*T*_0_ − 3*x*, where *x* = 1, 2, 3, 4, 5) °C. Finally, after cooling the samples to −60 °C, they were subjected to a heating phase, raising the temperature to 30 °C at a rate of 10 °C/min.

Crystallization Kinetics Test:

For the crystallization kinetics test, each sample was rapidly cooled to a specific temperature based on its crystallization temperature and held isothermally at that temperature for a duration of 30 min. Subsequently, the sample was heated to 30 °C at a rate of 10 °C/min. This procedure was repeated for each sample at various crystallization temperatures.

Dynamic Mechanical Properties Test:

Dynamic mechanical analysis (DMA850, TA Instruments, New Castle, DE, USA; sandwich shear clamp) was utilized for this assessment. Rubber sheets were processed on a vulcanizing machine to produce films with a thickness ranging from 0.8 to 1.0 mm. Square samples measuring 10 mm on each side were prepared using a cutter. Two of these sample sheets were then mounted onto the sandwich shear clamp, and the thickness of the samples was measured. Subsequently, the samples were cooled to −110 °C and maintained at this temperature for 5 min. The dynamic mechanical properties were examined by testing the transitions between −110 °C and 30 °C using a 0.15% strain, 1 Hz frequency, and a heating rate of 3 °C/min.

## 3. Results

### 3.1. Crystallization and Melting Behavior

**Figure 1:**
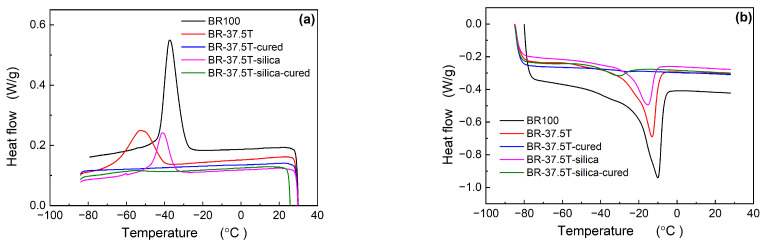
Crystallization (**a**) and melting (**b**) curves of Nd-BR/silica compounds.

Figure 1 presents the crystallization and melting curves for Nd-BR and their blends with silica, with detailed crystallization parameters outlined in Table 2. It is evident that pure Nd-BR exhibits a distinct crystallization peak during the cooling process, characterized by a crystallization peak temperature (*T*_c,peak_) of −37.44 °C. However, upon blending with 37.5 parts PHR TDAE oil, *T*_c,peak_ decreases notably to −52.7 °C, accompanied by a reduction in crystallization enthalpy (Δ*H*_c_) from 39.34 J/g to 22.99 J/g. Simultaneously, both the melting peak temperature (*T*_m,peak_) and melting enthalpy experience a decline. Upon crosslinking BR-37.5T, both the crystallization and melting peaks become indiscernible. The addition of fillers to BR-37.5T leads to a notable increase in *T*_c,peak_; however, due to the reduced Nd-BR content, both its Δ*H*_c_ and melting enthalpy Δ*H*_m_ remain lower compared to BR-37.5T. Following crosslinking, both Δ*H*_c_ and *T*_m,peak_ decrease, with the crystallization enthalpy diminishing from 15.01 J/g to 2.83 J/g. Additionally, a prominent cold crystallization behavior is observed during the heating process for BR-37.5T, its crosslinked variant, and the crosslinked version of BR-37.5T-silica.

**Figure 2:**
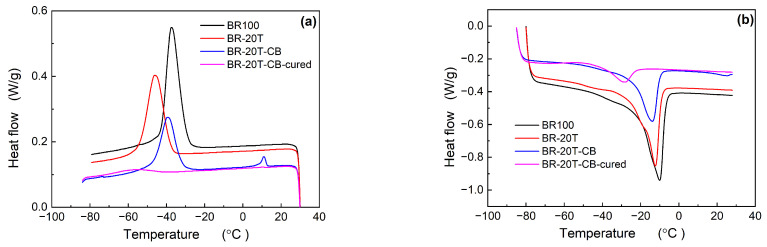
Crystallization (**a**) and melting (**b**) curves of Nd-BR/CB compounds.

Figure 2 depicts the crystallization and melting behaviors of Nd-BR and its composites with CB. Due to the lower TDAE oil content, both *T*_c,peak_ and *T*_m,peak_ of BR-20T are notably higher than those of BR-37.5T. Similar to the silica blends, the inclusion of CB results in an increase in *T*_c,peak_ while causing a decrease in *T*_m,peak_. Upon vulcanization, the rubber’s crystallization capability significantly diminishes, accompanied by the occurrence of cold crystallization.

As observed in the preceding discussion, the incorporation of TDAE oil and the initiation of the crosslinking reaction exert a substantial inhibitory influence on crystallization. Notably, a higher oil content leads to a more pronounced suppressive effect on crystallization. The crosslinking reaction restricts molecular mobility, thereby causing a noteworthy reduction in both crystallization temperature and enthalpy values [17,18].

**Figure 3:**
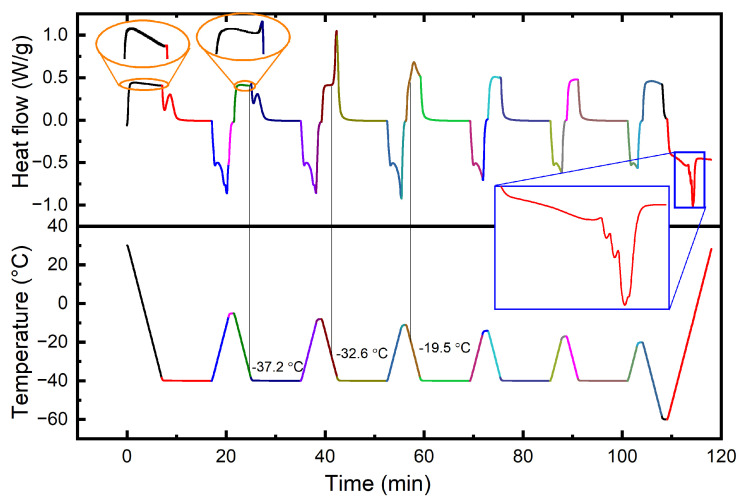
Crystallization curves of Nd-BR compounds with different maximum heating temperatures.

Conversely, fillers function as nucleating agents for crystallization, thereby elevating the crystallization temperature. To assess the impact of nuclei on crystallization, the effect of varying residual nuclei on the crystallization behavior of Nd-BR composites was investigated, as depicted in Figure 3. During the cooling of the sample to −40 °C, Nd-BR did not exhibit crystallization. However, significant crystallization behavior occurred during the isothermal hold at −40 °C. Subsequent heating to −5 °C led to the near-complete melting of crystals. However, upon subsequent cooling, crystallization commenced at −37.2 °C, likely attributed to the presence of residual nuclei acting as nucleating agents, thereby raising the crystallization temperature.

Further heating to −8 °C, while retaining some crystallization, followed by cooling, increased the initial crystallization temperature to −32.6 °C. Decreasing the melting temperature further to −11 °C resulted in the preservation of more unmelted crystals, thereby providing an abundance of nuclei for the cooling process. Consequently, this raised the initial crystallization temperature to −19.5 °C. Hence, it is evident that nuclei play a pivotal role in significantly elevating the crystallization temperature. The addition of fillers, exhibiting heterogeneous nucleation effects akin to those of nuclei [19,20], introduces crystallization nuclei, thereby resulting in a substantially higher crystallization temperature in the compounded rubber compared to rubber solely containing oil and lacking fillers.

### 3.2. Crystallization Kinetics

In accordance with crystallization kinetics principles, subjecting a sample to isothermal crystallization and establishing the relationship between the degree of crystallization and time through the Avrami equation provides insights into the crystallization kinetic parameters of Nd-BR [11,21]. Previous research findings indicate that during isothermal crystallization, the Avrami index *n* value for pure Nd-BR typically falls within the range of 2.5 to 2.7. Notably, at −50 °C, the crystallization rate exhibits a diminishing trend with increasing temperature, resulting in an extended half-crystallization time denoted as *t*_1/2_.

As outlined in Table 3, the *n* values for all rubber composites vary between 1.5 and 2.5. The introduction of TDAE oil results in reductions in both crystallization temperature and *n* value, with a trend of increasing *n* value as the crystallization temperature decreases. On the other hand, fillers elevate the crystallization temperature of the rubber, although the alterations in *n* value, when compared to rubber containing only oil, are minimal. Vulcanization further reduces the *n* value, placing it within the range of 1.5 to 2.0.

In accordance with the Lauritzen–Hoffman secondary nucleation theory [22], the crystallization rate is jointly determined by the nucleation rate and crystallization growth rate. Near the melting point, the crystallization rate is controlled by nucleation density, with higher temperatures leading to greater critical nucleation free energy barriers, resulting in slower nucleation rates. Conversely, at lower temperatures, the crystallization rate is governed by molecular diffusion velocity. As the temperature approaches the glass transition temperature (*T*_g_), the activation energy barrier for molecular motion increases, leading to a reduction in the crystallization growth rate. Consequently, the overall crystallization rate follows a bell-shaped curve between *T*_g_ and *T*_m_, with the peak crystallization rate typically occurring near (*T*_m_ + *T*_g_)/2.

For Nd-BR rubber, with *T*_g_ and *T*_m_ values of approximately −105 °C and −10.11 °C, respectively, its maximum crystallization temperature, denoted as *T*_c,max_, is estimated to be around −57.5 °C, consistent with the literature reports [7]. Table 3 reveals that for temperatures above −56 °C, *t*_1/2_ increases with rising temperature, whereas below −56 °C, *t*_1/2_ decreases as the temperature increases. This observation suggests that while TDAE oil, fillers, and crosslinking reactions exert substantial effects on crystallization, the temperature ranges governed by nucleation and growth control remain unaltered.

According to the Arrhenius equation, the crystallization rate can be correlated with temperature. Combining the relevant formula t1/2=(ln2k)1/n, we can derive the expression $k=\frac{ln2}{t_{1/2}^{n}}=Aexp(\frac{-E_{a}}{RT})$, where *A* is a constant and *E*_a_ represents the crystallization activation energy. Rearranging this equation yields $lnt_{1/2}=\frac{ln(\frac{\ln2}{A})}{n}+\frac{E_{a,t(1/2)}}{nRT}$. This equation allows us to calculate the crystallization activation energy *E*_a_ using the half-crystallization time, as presented in Table 2. The crystallization activation energy for pure Nd-BR is determined to be −264.3 kJ/mol. However, the introduction of TDAE oil results in a decrease in this value, with BR-20T and BR-37.5T showing values of −116.8 kJ/mol and −48.1 kJ/mol, respectively. Fillers, serving as nucleating agents, raise the crystallization temperature while simultaneously reducing the crystallization activation energy. For instance, the value for BR-37.5T-silica is −239.1 kJ/mol, while for the CB system, it is −224.9 kJ/mol. Crosslinking substantially restricts molecular mobility, diminishing the temperature’s impact on such mobility [16], leading to a reduced absolute value of the crystallization activation energy. The value for the silica composite stands at −239.1 kJ/mol, but post crosslinking, it drops to −155.8 kJ/mol.

**Figure 4:**
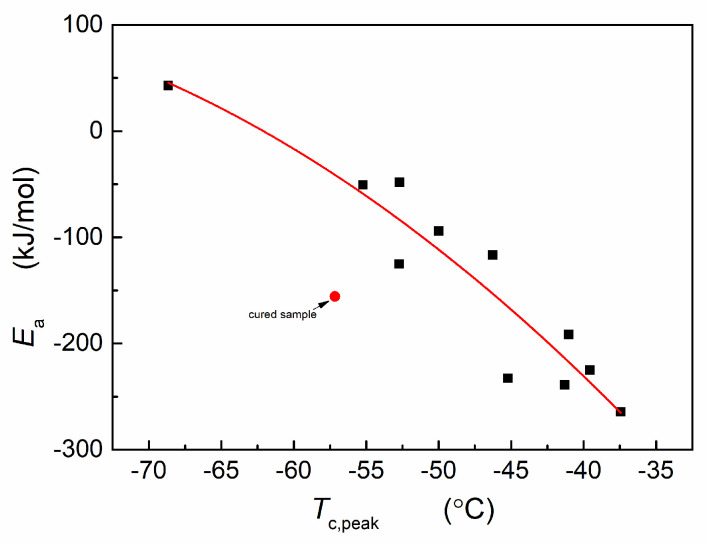
Relationship of *E*_a_ with *T*_c,peak_. ▪, non-crosslinked samples; ●, cured sample.

Crystallization activation energies were determined across various temperature intervals for different samples. Comparing the activation energy with the crystallization temperature of the sample reveals that the crystallization activation energy increases as the crystallization temperature decreases. Below −56 °C, the crystallization activation energy transitions from a negative to a positive value, and the crystallization rate escalates with rising temperature. Notably, for the crosslinked silica system, despite having a crystallization temperature close to that of BR-37.5T, the restriction on molecular mobility due to crosslinking reduces temperature dependence and significantly elevates crystallization activation energy. After combining the results in our previous study [16], it is concluded that oil and fillers primarily influence the nucleation temperature of Nd-BR, while the crystallization activation energy is predominantly correlated with the crystallization temperature, as is shown in Figure 4. On the other hand, crosslinking reaction restricts the movement of molecules and retards the crystallization, resulting in the decrease in the absolute value of activation energies.

The melting point represents the temperature at which a polymer crystallite melts. The relationship with the thickness of the crystallite can be expressed as $T_{m}=T_{m}^{0}(1-\frac{2\sigma_{e}}{l∆h})$, where *T*_m_ and $T_{m}^{0}$ denote the melting points of the crystallite at thickness *l* and infinite thickness, respectively. ∆h represents the enthalpy of fusion per unit volume, and $\sigma_{e}$ is the surface energy. As inferred from Table 2, the melting points of samples consistently increase with a rise in crystallization temperature. The equilibrium melting point, $T_{m}^{0}$, can be obtained from the intersection of the *T*_m,peak_~*T*_c_ curve with the line *T*_m_ = *T*_c_. Based on data presented in Table 3, the $T_{m}^{0}$ for the Nd-BR/silica composite is approximately −5.23 °C, which decreases to −10.16 °C post vulcanization. Conversely, the $T_{m}^{0}$ for the Nd-BR/CB composite stands at about −3.13 °C, dropping to −21.6 °C after vulcanization. As reported, CB exerts a robust interfacial adsorption effect with rubber molecules, imposing a more substantial restriction on molecular chain motion than silica. However, the high adsorption ability of curatives and the high acidity of the silica surface due to hydroxyl result in poor cure characteristics, leading to a lower cure rate and lower yield of crosslinks during vulcanization [23]. And the higher concentration of TDAE oil leads to the swelling of rubber and reduces trapped entanglement of the crosslinked network. Therefore, post vulcanization, the Nd-BR/silica composite presents a higher equilibrium melting point.

### 3.3. Dynamic Mechanical Properties

**Figure 5:**
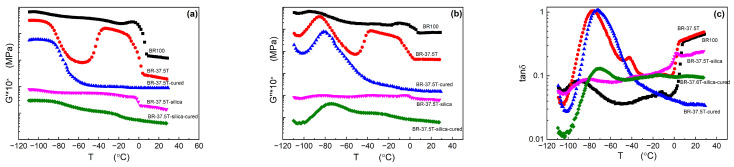
DMA curves of Nd-BR compounds. (**a**) Storage modulus *G*′, shifted vertically by 10^×^; (**b**) loss modulus *G*″, shifted vertically by 10^×^; (**c**) loss tangent, tan *δ*.

DMA offers valuable insights into the mechanical behavior of materials influenced by crystallization [5,24]. The DMA curve of the Nd-BR/silica composite system is presented in Figure 5. It is discernible that the glass transition region for Nd-BR occurs within the temperature range of −100 °C to −60 °C. Due to the crystallization of BR100 during cooling, it predominantly retains a crystalline state after the glass transition, resulting in a modest reduction in the storage modulus (*G*′) while keeping the loss factor (tan *δ*) low. As the temperature nears 0 °C and the crystals melt, a significant decrease in *G*′ and a surge in tan *δ* are observed. At room temperature, the *G*′ of pure Nd-BR is about 0.2 MPa after melting of Nd-BR.

Incorporating 37.5 PHR TDAE oil into Nd-BR introduces dilution effects, increasing the spacing between polymer chains. This leads to a reduced crystallization temperature and rate, causing the sample to remain amorphous during rapid cooling. Consequently, a sharp transition from a glassy to a rubbery state occurs during the glass transition, resulting in a substantial drop in modulus and an increase in tan *δ*. However, when the temperature exceeds −60 °C, cold crystallization occurs (as evidenced by Figure 1b), causing a sudden rise in both *G*′ and the loss modulus (*G*″). Near 0 °C, the melting of crystals prompts a decrease in modulus and a peak in tan *δ*. Due to the swelling effect, the *G*′ of Nd-BR with 37.5 PHR TDAE oil is only about 0.1 MPa, which is much lower than that of pure Nd-BR.

Upon crosslinking BR-37.5T, *G*′ increased obviously to 0.187 MPa at 25 °C. Meanwhile, the glass transition becomes more pronounced, and its temperature shifts to higher temperature, as reflected in the *G*′ and tan *δ* curves. Additionally, due to restricted molecular motion post crosslinking, the ability to crystallize diminishes. The sample lacks nucleation during heating, eliminating the phenomenon of cold crystallization, with the modulus gradually decreasing with temperature.

Fillers serve as reinforcing agents and nucleating agents at the same time, resulting in elevating the crystallization temperature and rate. Therefore, the BR-37.5T-silica sample can crystallize rapidly during cooling, exhibiting a weak glass transition with a relatively high modulus in the crystalline state. At 0 °C, as the crystals melt, tan *δ* increases. After vulcanization of the composite rubber, *G*′ increased from 0.4 MPa to 2.2 MPa after crosslinking reaction at high temperature due to crosslinked network, but it decreased from 7.6 MPa to 4.8 MPa at −40 °C due to lower crystallization capabilities. The nucleating effect of the filler facilitates limited crystallization during cooling. The modulus within the glass transition region is noticeably reduced due to lower crystallinity, and the transition’s tan *δ* becomes higher, shifting to a higher temperature. Concurrently, minimal crystallization leads to a plateau in modulus post transition, with melting occurring around −20 °C, causing a decline in modulus.

From the aforementioned observations, it is evident that whether it is pure Nd-BR or its composites, crystallization is possible at low temperatures. Fillers, acting as nucleating agents, enhance the rubber’s crystallization capabilities, resulting in an increased modulus and a harder material, which is undesirable for low-temperature applications. While TDAE oil and crosslinking can inhibit the extent of crystallization and lower the material’s low-temperature modulus, it remains challenging to entirely eliminate low-temperature crystallization in Nd-BR composites.

## 4. Conclusions

In the realm of rare-earth butadiene rubber, the intricate interplay of various additives and crosslinking reactions exerts a profound influence on crystallization behavior. Specifically, the introduction of TDAE oil and the subsequent crosslinking process engender a noteworthy elevation in the glass transition temperature, concurrently exerting a substantial suppressive effect on crystallization. This dual impact results in an elevated crystallization activation energy and a commensurate reduction in the equilibrium melting point, with the crosslinking network, in particular, being responsible for a pronounced decline in the latter. Whether within compounded rubber or its vulcanized counterpart, fillers consistently assume the role of nucleating agents, effectively raising the crystallization temperature and expediting the crystallization rate.

However, it is noteworthy that the combined influences of additives, fillers, and crosslinking appear to uphold the intrinsic correlation between crystallization activation energy and temperature. And oil and fillers primarily influence the nucleation temperature of Nd-BR, while the crystallization activation energy is predominantly correlated with the crystallization temperature. Notably, the crystallization activation energy exhibits an upward trajectory as the crystallization temperature decreases, eventually transitioning from a negative to a positive value at approximately −56 °C. Furthermore, the dynamic mechanical properties of Nd-BR remain intimately intertwined with crystallization. This connection results in an augmentation of the modulus and a simultaneous reduction in the loss factor following crystallization, accompanied by a significant constraint on the glass transition.

## Figures and Tables

**Table 1:** Rubber formulation of compounds based on carbon black and silica, respectively.

	Nd-BR	N330	TDAE Oil	ZnO	SA	Sulfur	CBS			
BR100	100									
BR-20T	100		20							
BR-20 PHR oil-CB *	100	50	20	5	2	1.5	0.9			
	Nd-BR	N234	Silica	TESPT	TDAE oil	ZnO	SA	Sulfur	CBS	DPG
BR-37.5T	100				37.5					
BR-37.5 PHR oil *	100				37.5	4	2	1.5	1.8	1.5
BR-37.5 PHR oil-silica *	100	5.6	70	5.6	37.5	4	2	1.5	1.8	1.5

Note: * samples are also tested after vulcanization.

**Table 2:** Crystallization and melting parameters of Nd-BR compounds.

	*T*_c,peak_ (°C)	Δ*H*_c_ (J/g)	*T*_m,peak_ (°C)	Δ*H*_m_ (J/g)	*T*_c,cold_ (°C)	Δ*H*_c,cold_ (J/g)	$T_{m}^{0}$ (°C)	*E*_a_ (kJ/mol)
BR100	−37.44	39.34	−10.11	43.75	/	/	−0.135	−264.3
BR−37.5T	−52.70	22.99	−13.14	25.83	−57.72	0.987	/	−48.1
BR-37.5 PHR oil-cured			−26.33	0.41	−48.85	0.201		/
BR-37.5 PHR oil-silica	−41.30	15.01	−15.23	17.07	/	/	−5.23	−239.1
BR-37.5 PHR oil-silica-cured	−57.16	2.83	−30.39	4.10	−53.85	0.626	−10.16	−155.8
BR-20T	−46.27	30.66	−12.33	32.09	/	/	/	−116.8
BR-20 PHR oil-CB	−39.55	21.52	−13.94	22.16	/	/	−3.13	−224.9
BR-20 PHR oil-CB-cured	−56.38	3.73	−28.42	6.27	−52.15	1.279	−21.60	≥−56 °C: −29.4 ≤−56 °C: 9.97

**Table 3:** Crystalline parameters of Nd-BR/TDAE oil compounds.

		*T*_c_ (°C)	*T*_m,peak_ (°C)	*t*_1/2_ (s)	*n*	*lgk*
Silica system	BR-20 PHR oil	−38	−14.58	198.48	1.9662	−4.8374
		−40	−14.00	186.42	2.0081	−4.7249
		−42	−13.66	161.52	1.9004	−4.5535
		−44	−13.46	143.76	2.1340	−4.8427
		−46	−13.38	134.70	2.2232	−4.8913
	BR-20 PHR oil-silica	−26	−11.90	334.44	2.1798	−5.6569
		−28	−12.63	209.04	2.1995	−5.0839
		−30	−13.32	122.40	2.2554	−5.6049
		−32	−13.95	87.60	2.3141	−4.7527
		−34	−14.48	56.58	2.4124	−4.4582
	BR-20 PHR oil-silica-cured	−38	−21.95	292.98	1.55	−4.0175
		−39	−22.42	246.90	1.63	−4.176
		−40	−22.80	191.70	1.73	−4.2012
		−41	−23.24	166.50	1.80	−4.2557
		−42	−23.66	131.10	1.89	−4.2271
Carbon black system	BR-20 PHR oil	−32	−12.36	267.00	1.97	−5.0068
		−34	−12.90	203.70	1.95	−4.7291
		−36	−12.64	154.80	1.98	−4.5457
		−38	−11.96	127.50	2.24	−4.9465
		−40	−11.70	102.54	2.36	−4.9469
	BR-20 PHR oil-CB	−24	−10.56	594.78	1.95	−5.5629
		−26	−11.49	352.50	1.95	−5.1218
		−28	−12.22	236.52	1.96	−4.9977
		−30	−12.93	152.10	2.20	−4.8771
		−32	−13.46	100.92	2.31	−4.8175
	BR-20 PHR oil-CB-cured	−52	−28.02	664.32	1.71	−4.9868
		−54	−28.55	614.58	1.78	−5.1127
		−56	−29.01	564.42	1.93	−5.4321
		−58	−29.42	573.60	1.91	−5.3886
		−60	−29.72	595.44	1.96	−5.5483

## Data Availability

The data that support the findings of this work are available from the corresponding author upon reasonable request.
